# Exploring Droughts and Floods and Their Association with Cholera Outbreaks in Sub-Saharan Africa: A Register-Based Ecological Study from 1990 to 2010

**DOI:** 10.4269/ajtmh.17-0778

**Published:** 2018-03-05

**Authors:** Andreas Rieckmann, Charlotte C. Tamason, Emily S. Gurley, Naja Hulvej Rod, Peter Kjær Mackie Jensen

**Affiliations:** 1 University of Southern Denmark, Odense, Denmark;; 2 University of Copenhagen, Copenhagen, Denmark;; 3 Johns Hopkins Bloomberg School of Public Health, Baltimore, Maryland

## Abstract

Cholera outbreaks in Africa have been attributed to both droughts and floods, but whether the risk of a cholera outbreak is elevated during droughts is unknown. We estimated the risk of cholera outbreaks during droughts and floods compared with drought- and flood-free periods in 40 sub-Saharan African countries during 1990–2010 based on data from Emergency Events Database: the Office of Foreign Disaster Assistance /Centre for Research on the Epidemiology of Disasters International Disaster Database (www.emdat.be). A cholera outbreak was registered in one of every three droughts and one of every 15 floods. We observed an increased incidence rate of cholera outbreaks during drought periods (incidence rate ratio [IRR] = 4.3, 95% confidence interval [CI] = 2.9–7.2) and during flood periods (IRR = 144, 95% CI = 101–208) when compared with drought/flood-free periods. Floods are more strongly associated with cholera outbreaks, yet the prevalence of cholera outbreaks is higher during droughts because of droughts’ long durations. The results suggest that droughts in addition to floods call for increased cholera preparedness.

## INTRODUCTION

Half of the world’s reported cholera cases occur in sub-Saharan Africa, which is also the region with the highest case-fatality rates of cholera.^1^ Single-country studies and one review of cholera outbreaks in Africa have linked cholera outbreaks to both droughts and floods.^2–4^ Whereas floods are a recognized risk factor of cholera transmission by the World Health Organization (WHO), droughts are not explicitly regarded as a risk factor in guidelines.^5,6^ If the risk of a cholera outbreak is increased during droughts in addition to floods in Africa, established cholera preparedness procedures should apply to droughts and floods.

The casual chain to cholera outbreaks is complex and depends on many socioeconomic and environmental factors, specifically water, hygiene, and sanitation.^7^ It is estimated that 68% of the population in sub-Saharan Africa has access to an improved drinking water source heterogeneously distributed,^8^ and the quality of these improved sources are largely unknown. As of 2012, only 30% of the population in sub-Saharan Africa was estimated to have had access to improved, non-shared sanitation facilities.^8^ Understanding the risks for cholera outbreaks during droughts and floods allows for better preparedness, which can reduce morbidity and mortality. However, no multi-country study has systematically assessed the risk for cholera outbreaks during both droughts and floods. This insight is particularly relevant in the light of climate changes that are projected to increase the duration and severity of droughts and the severity and frequency of floods in the tropical belt of the world.^9^ Droughts decrease the level of water availability and thereby the hygiene in a population, which we hypothesized increased the risk of diarrheal disease outbreaks, including cholera.^10^ In this explorative study, we aimed to estimate the risk of cholera outbreaks during high-impact droughts and floods in sub-Saharan Africa.

## METHODS

This is a register-based country-level ecological study from 1990 to 2010.

### Data sources.

**Figure 1.**
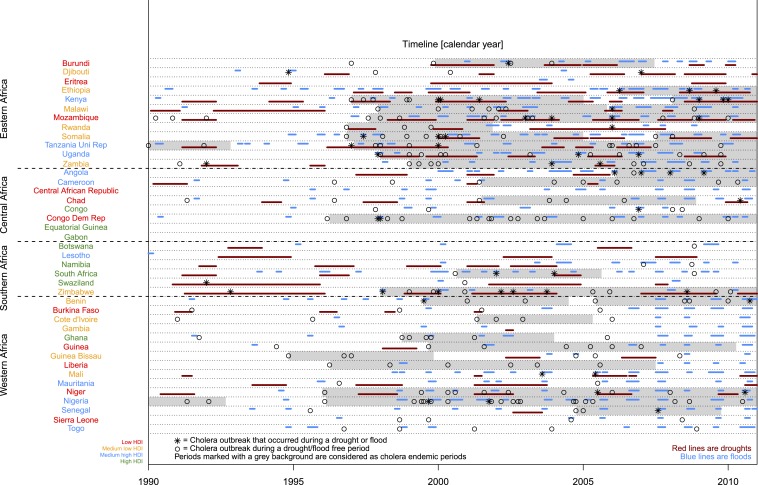
Overview of registered droughts, floods, and cholera outbreaks in sub-Saharan Africa from 1990 through 2010. This figure appears in color at www.ajtmh.org.

We obtained data on droughts, floods, and cholera outbreaks registered from 1990 through 2010 in EM-DAT: International Disaster Database—www.emdat.be, Université Catholique de Louvain, Brussels.^11^ Events are registered according to the definitions by EM-DAT on epidemics (cholera outbreaks being specifically termed “cholera”), droughts, and floods (see Supplemental Table 1 for disaster definitions) with dates of beginning and end and specific location. EM-DAT systematically comprises events where 10 people die, or 100 people are affected, or that result in a state of emergency or call for international assistance from sources as United Nations agencies, governmental and nongovernmental agencies (NGO), insurance companies, research agencies, and press agencies. Thus, events are considered of high impact/severe because of the inclusion criteria. Epidemics are primarily reported by WHO. We analyzed 41 sub-Saharan African mainland countries (Figure 1).

### Cholera outbreaks.

A cholera outbreak is defined by isolation of *Vibrio cholerae of O1 or O139 serogroups* from one case. Subsequently, the case definition is relaxed.^12^ World Health Organization recognizes underreporting of cholera outbreaks due to lack of detection in remote areas and political expediency of nations avoiding the potential economic impacts and political implications of releasing such information.^13^ Hence, we investigated events registered as “acute diarrheal syndrome” and “acute watery diarrhea” in EM-DAT for being potential cholera outbreaks. If a reputable source (Médecins Sans Frontières, promedmail.org, reliefweb.org, the International Federation of Red Cross and Red Crescent Societies, WHO/Pan American Health Organization, United Nations Children’s Fund, Integrated Regional Information Networks News, and peer-reviewed journals) reported a cholera outbreak in the same region or district within the same country and during the same time frame as a diarrheal outbreak, the event was recoded to a cholera outbreak.

### Missing dates and affected populations for droughts and floods.

Missing dates for droughts and floods were searched for through reputable sources (Supplemental Table 1) and otherwise imputed using the respective start and end of the countries’ dry and wet season based on annual rainfall data on subnational levels from http://www.worldweatheronline.com. If both start and end dates were missing for droughts, we assumed it lasted from the beginning of this year’s dry period during the wet season and until the end of the next dry season. For droughts and floods missing the number of affected people, we used the respective medians (single imputed).^14^

### Analysis.

Because of the slow onset of droughts, EM-DAT registers drought start dates by month only.^15^ We therefore used months as the time unit in our analyses as they were the most precise common time unit available for the included events (Supplemental Table 2). We reasoned that cholera outbreaks may not be registered until weeks after the primary case of cholera and cholera outbreaks immediately following a drought or flood could still be related to the respective disaster. Therefore, the exposure time for each flood and drought was calculated from the beginning of the respective disaster’s start month to the end of its registered end month, plus one additional month. A sensitivity analysis was performed where no additional month was added to the end of the flood or drought periods.

We classified all cholera outbreaks according to whether they began during a drought, flood, or a drought- and flood-free period. Cholera outbreaks were considered to have begun during a drought or flood if the respective drought or flood was ongoing at the initiation of the cholera outbreak and in the same region or state reported in the EM-DAT register. If the registered location names or spellings were indeterminate, we checked the locations visually using Google Maps to see if the events in fact occurred in the same area enabling us to match locations on different spatial scales.

Next, we calculated incidence rates of cholera during drought, flood, and drought/flood-free periods. Because most droughts and floods did not affect an entire country, we weighted the rates accounting for the affected population by the total country population (Supplemental Table 3 and illustrated in Supplemental Figure 1). The affected population is defined by EM-DAT as the sum of the injured, affected, and left homeless after a disaster. As a sensitivity analysis to adjust for time-fixed confounding between countries, we applied the self-controlled case series method,^16^ which is inspired by the case crossover design using conditional Poisson regression of the countries with cholera outbreaks only.

Regional differences and countries’ capacities (operationalized with the human development index [HDI]) could be confounders. We expected that regional climate could influence the occurrence of floods and droughts, whereas endemicity and cross-border spread could affect the regional incidence of cholera outbreaks, so we performed a sub-analysis of eastern-, western-, central-, and southern Africa. Also, because disasters are known to disproportionately affect people with low socioeconomic status and recurring disasters can result in more poverty and lower socioeconomic status,^17^ we speculated that socioeconomic differences (operationalized with HDI) may affect countries’ capabilities to prevent cholera outbreaks and disasters. To investigate heterogenic effects by HDI, we stratified the included countries by their respective HDI values in 2010^18^ (Figure 1).

Because cholera peaks may follow seasonal patterns in endemic areas,^12^ we reasoned that we might find an increased association between cholera outbreaks and droughts and floods in endemic areas compared with non-endemic areas. Therefore, we explored if endemicity of cholera was an effect modifier to the association between droughts and floods and cholera outbreaks by separately analyzing endemic and non-endemic periods. We used a definition of endemic periods already used by Ali et al.^19^ as a 5-year period of time in a single country during which cholera outbreaks were registered during three or more years (Supplemental Table 1). The definition origins from work by the WHO Strategic Advisory Group of Experts on Vaccines and Immunization.^20^ We searched for cholera outbreaks before 1990 in EMDAT and cholera outbreaks after 2010 in Google to fully apply the definition.

## RESULTS

Two hundred and forty-eight outbreaks were registered as cholera outbreaks. Thirty-nine outbreaks were registered as “acute diarrheal syndrome” and “acute watery diarrhea,” of which 28 were confirmed via location and time period by reputable sources (see Supplemental Table 1) to be cholera outbreaks; thus, a total of 276 cholera outbreaks were registered during the study period. Four of 276 of the cholera outbreaks were missing start dates. We were able to confirm the start month of four of the outbreaks through online epidemiological records (WHO and Reliefweb) and the remaining three were excluded. We imputed the missing start month of the 15% of registered droughts (8/118) and the missing end month for 72% of droughts (85/118). The mean length for droughts with no missing versus all droughts including imputed values was 9 and 14 months, respectively. No floods were missing data for the start month. With regard to the number of affected individuals from a drought or flood, we imputed the median value being 750,000 people for 14 (11%) of the droughts and the median value being 10,000 people for 33 (6%) of floods.

During the 21 years of follow-up within the 41 sub-Saharan countries (861 country-years), a total of 276 cholera outbreaks and 118 drought and 515 flood disasters occurred. Two countries, Gabon and Equatorial Guinea, had no registered cholera outbreaks, droughts, or floods in the EM-DAT database during the time of study (Figure 1). The median length of droughts was 1.2 years (inter quartile range [IQR]: 0.55) and floods was 0.17 years (IQR: 0.04). Figure 1 shows a temporal visualization of the analyzed data.

A single cholera outbreak typically occurred in multiple municipalities within one country. As such, certain cholera outbreaks were registered as starting during both droughts and floods within the same country. Twenty-five cholera outbreaks began during only drought, 24 cholera outbreaks began during only flood, and 10 cholera outbreaks were registered as beginning during both a flood and a drought. The remaining 217 cholera outbreaks (79% of all cholera outbreaks) began during drought/flood-free periods.

The number of cholera outbreaks per drought was 4.5 (95% confidence interval [CI] = 2.9–6.9) times larger than for floods (0.30 versus 0.06) (Table 1). The incidence rate of cholera outbreaks that began during drought periods was 1.1 outbreaks per weighted country-year and 38 outbreaks per weighted country-year during flood periods. The incidence rate of cholera outbreaks during drought/flood-free periods was 0.26 times per weighted country-year. The incidence rate of cholera outbreaks during drought periods compared with drought/flood-free periods was 4.3 times higher (95% CI = 2.9–7.2), whereas the incidence rate ratio of cholera outbreaks during flood periods compared with drought/flood-free periods was 144 times higher than drought/flood-free periods (95% CI = 101–208) (Table 1).

**Table 1:** Association between cholera outbreaks occurring during droughts, floods, and drought/flood-free periods

		Cholera outbreaks that began during the disaster	Number of disasters	Risk (cholera outbreak/disaster)	Risk time (weighted country-years)	Incidence rate (cholera outbreaks/weighted country-years)	Cholera outbreaks beginning during drought/flood-free periods	Drought/flood-free time (weighted country-years)	Incidence rate (cholera outbreaks/weighted country-years)	Incidence rate ratio (95% confidence interval)
Drought		35	118	0.30	31.1	1.13	217	829.0	0.26	4.3 (2.9–7.2)
Region	Eastern Africa	24	64	0.38	15.7	1.53	79	235.9	0.33	4.6 (2.9–7.2)
	Central Africa	1	10	0.10	0.4	2.44	40	167.5	0.24	10.2 (1.4–74.3)
	Southern Africa	8	21	0.38	11.8	0.68	13	114.1	0.11	6.0 (2.5–14.4)
	Western Africa	2	23	0.09	3.3	0.61	85	311.5	0.27	2.2 (0.6–9.1)
HDI*	Low	6	31	0.19	6.5	0.92	74	224.3	0.33	2.8 (1.2–6.4)
	Medium low	16	37	0.43	11.1	1.44	49	219.6	0.22	6.5 (3.7–11.4)
	Medium high	9	32	0.28	5.9	1.53	68	204.0	0.33	4.6 (2.3–9.2)
	High	2	13	0.15	6.3	0.32	17	161.5	0.11	3.0 (0.7–13.1)
Flood		34	515	0.07	0.9	37.9	217	829.0	0.26	144 (101–208)
Region	Eastern Africa	16	219	0.07	0.5	33.8	79	235.9	0.33	101 (59–173)
	Central Africa	8	81	0.10	0.1	142.1	40	167.5	0.24	595 (278–1,272)
	Southern Africa	3	47	0.06	0.1	23.9	13	114.1	0.11	210 (60–737)
	Western Africa	7	168	0.04	0.2	28.8	85	311.5	0.27	106 (49–228)
HDI*	Low	7	122	0.06	0.2	38.0	74	224.3	0.33	115 (53–250)
	Medium low	9	137	0.07	0.3	26.3	49	219.6	0.22	118 (58–240)
	Medium high	15	172	0.09	0.1	138.8	68	204.0	0.33	416 (238–728)
	High	2	58	0.03	0.2	11.9	17	161.5	0.11	113 (26–488)

HDI = human development index. Relative risk of cholera outbreak during droughts compared with floods: 4.5 [2.9–6.9]. Incidence rate ratio of cholera outbreaks beginning during floods compared with droughts: 34 [20–54]. *P* values of incidence rate ratio derived by *z*-test.

* Somalia is excluded because of missing HDI information.

The self-controlled case series analysis that controls for time-fixed confounding gave similar estimates as our main analysis; incidence rate ratio (IRR) of a cholera outbreak during droughts versus drought/flood-free periods was 5.37 (95% CI = 3.55–8.11) and for floods versus drought/flood-free periods was 145 (95% CI = 97–217).

The sensitivity analysis, where no extra month was added to each disaster length, yielded similar results; droughts: an IRR of 4.4 (95% CI = 3.1–6.3) and floods: an IRR of 216 (95% CI = 148–314) when compared with drought/flood-free periods.

The rate of cholera outbreaks that began during droughts and floods compared with the rate during drought/flood-free periods was significantly higher within all four regions of sub-Saharan Africa except during droughts in western Africa (Table 1). The stratification by HDI showed significant associations between droughts, floods, and cholera outbreaks in all categories except the highest HDI group for droughts (Table 1).

Approximately, one-fourth of the entire study period was considered cholera endemic (Figure 1). Although droughts and floods were statistically significantly associated with an increased risk of cholera outbreaks in both endemic and non-endemic periods compared with drought/flood-free periods, we observed a slightly higher risk of cholera outbreaks during floods in non-endemic periods compared with endemic periods (test for a different IRR, *P* value = 0.10) (Supplemental Table 4).

## DISCUSSION

Floods were—as expected—associated with an extraordinarily high incidence rate of cholera outbreaks during floods compared with flood/drought-free periods (IRR = 144 [95% CI = 101–208]). However, droughts too were associated with a higher IRR of 4.3 (95% CI = 2.9–7.2) compared with flood/drought-free periods. As droughts naturally last longer than floods and affect a larger area, a higher proportion of droughts than floods may be expected to overlap with cholera outbreaks; we found that a cholera outbreak began during 30% of all registered droughts; this is more than four times higher than the proportion of cholera outbreaks that began during registered floods. This understanding that cholera outbreaks are often seen during droughts could potentially improve preparedness during droughts and prevent cholera deaths; however, it is also important to emphasize that the vast majority of cholera outbreaks (217/276) began during drought/flood-free periods, whereas 35 began during droughts and 34 began during floods.

One could expect an increased association between cholera outbreaks and droughts and floods in endemic periods when compared with non-endemic periods. Contrarily, we found that floods were associated with a higher risk of cholera outbreaks during non-endemic periods compared with endemic periods—although flood and droughts in both endemic and non-endemic periods were associated with an increased incidence rate of cholera outbreaks. This could be due to decreased susceptibility of the population to cholera in the endemic periods.^21^

### Strengths and limitations.

The advantage of EM-DAT is their defined inclusion criteria (see Introduction) as the database aim for scientific research and for the development community.^22^ However, one limitation of EM-DAT is that reported cholera outbreaks may not have been laboratory confirmed as *Vibrio cholerae*, and we are unable to discern which were confirmed. It is possible that outbreaks caused by other pathogens were included in our analysis, which could reduce our power to detect real associations with cholera outbreaks. There are reports in the published literature of suspected outbreaks of cholera being diagnosed as other pathogens,^23,24^ although these are rare suggesting that cholera outbreaks were unlikely to be commonly misclassified in EM-DAT. Many small and medium disasters go unnoticed, which potentially could be of high cumulative impact.^25^ This narrows our analysis to only high-impact droughts, floods, and cholera outbreaks. Some underreporting of cholera outbreaks may be present, but our results seemed robust across geographical and HDI strata, which does not indicate a systematic bias.

We increased the accuracy of our data by manually confirming locations of cholera outbreaks and corresponding floods and droughts. Because some disasters were recorded as overlapping during the same place and time, some double-counted risk time was subsequently subtracted from drought/flood-free periods estimating conservative estimates. Our country weights were applied to handle the fact that countries are only partly affected by droughts and floods at a time; however, countries with small populations would be weighted more than countries with large population if affected by a similar disaster. This would favor the association between droughts and floods and cholera outbreaks in countries with small populations over countries with large populations for the combined estimate.

Human development index was speculated to affect both the capacity to respond to natural disasters and prevent cholera outbreaks; when stratifying by HDI, we observed—in contrary to a dose–response relationship—the strongest associations in the middle HDI groups. Importantly, the causality may be reversed and countries’ HDI may itself be affected by natural disasters and cholera outbreaks.

In acute disasters, such as floods and droughts, the international attention and presence of national organizations and international NGOs with cholera-testing resources may increase the probability of cholera outbreaks being reported and thereby create a spurious positive association between droughts, floods, and cholera outbreaks. This may have overestimated the association we found between cholera outbreaks and both droughts and floods. The fact that Equatorial Guinea and Gabon in central Africa did not have any registered droughts, floods, or cholera outbreaks made us consider having made an ecological fallacy by analyzing heterogenic countries with much risk-free time in drought/floods-free periods together with countries with high rates of cholera outbreaks both during and not during droughts and floods; however, the association persisted when analyzing each region separately. Also and more importantly, when we conducted the self-controlled case series analysis adjusting for time-fixed confounding, the similar results to the main results were reached. We cannot exclude that the use of separate sources respectively for weighting risk time using the affected population over the total population and for matching disasters and cholera outbreaks using text-based descriptions of locations may have influenced our estimates. Last, we did not account for the number of cholera cases per cholera outbreak.

Drought durations are difficult to measure because of their slow onset. According to Below et al.,^15^ droughts are included in the EM-DAT when the drought-related losses start, which can be much later than the droughts initiate. This could mean that some cholera outbreaks that began during droughts were registered as occurring during drought/flood-free periods, which would underestimate the association. Also, we did not account for mobile populations in our country-year weight under the assumption that the fluctuation of refugees only affects the country population marginally. To triangulate our data, we made a spot check with the cholera outbreaks in Togo registered at ReliefWeb and ProMED-mail, and the EM-DAT database included more cholera outbreaks than ReliefWeb and ProMED-mail. Our approach to handle missing data indicated an increase in the mean length of droughts, which could indicate an overestimation of the drought periods. This would lead to a conservative estimate.

### Comparisons with other studies.

World Health Organization highlights that although floods are associated with a risk of infectious disease, few lead to diarrheal disease outbreaks, which is consistent with our finding that cholera outbreaks began during only one out of every 14 floods.^5^ Reviews of cholera outbreaks have found that the outbreaks were often attributed to heavy rains^26^ and that although cholera outbreaks are registered during floods, they have been reported during drought situations as well.^2^ Also supportive of our findings is a climate modeling study from Bangladesh that compared climactically “average” seasons with seasons of severe droughts and severe floods and concluded that both severe drought seasons and severe flood seasons corresponded with an increase in cholera incidence.^27^ In early 1950s, the largest cholera outbreaks in India were noted to occur subsequent to failed monsoons and during drought-induced famines.^28^ In Mali during the 1980s, cholera outbreaks regularly began during major droughts.^3^ A cholera outbreak in Zimbabwe followed a severe drought and large displacement from Mozambique in 1992^29^ and was identified as one of the five important factors for the cholera outbreak.^4^

### Mechanisms.

Cholera may be person-to-person transmitted or acquired from aquatic reservoirs (e.g., lakes and oceans) of *Vibrio cholerae*.^2^ Displaced populations because of natural disaster may be responsible for introducing cholera to an area, or an influx of aid workers during floods and droughts could import cholera to these areas, as was witnessed in Haiti in 2010.^30^

Floods may contribute to cholera outbreaks in a number of ways. Floodwaters can overflow sanitation systems and contaminate the environment and water sources. In addition, they may impede access to safe water sources or sanitation facilities. In a review of ProMED reports, the most commonly cited risk factor for cholera outbreaks in southern and western Africa was heavy rainfall and flooding.^26^ In Bangladesh, both high and low rainfall have been associated with an elevated risk of cholera.^31^

There are also a number of potential mechanisms that could accelerate cholera transmission during droughts specifically through fomites due to a lack of water for hygiene.^32,33^ Studies indicate that water quantity is a key factor in preventing fecal–oral diseases such as cholera. A review of 67 studies of diarrheal morbidity and mortality indicated that reductions in diarrhea-related morbidity were associated with both access to sufficient household water (27%) and improved hygiene (33%).^34^ Limited fuel for cooking, long-term storage of food, and lack of acidic ingredients are also elements that have previously been suggested as conditions that could result in a higher risk of cholera.^3^

In addition, both droughts and floods may have several mechanisms in common that could affect the risk of cholera outbreaks. For example, population displacement can lead to crowding—potentially exacerbating sufficient water, hygiene, and adequate sanitation concerns—and resulting in more human-to-human interaction, increasing the risk of fecal–oral spread.^35^ Limited access to food and the ability to cook, which is likely in floods and droughts, can result in malnutrition and lower stomach acid increasing the risk of infection.^36^

## CONCLUSION

Our analysis suggests that countries in sub-Saharan Africa can expect to see a cholera outbreak in one out of every three droughts. This is more than four times as often as they would occur during floods. Our findings indicate that droughts in addition to floods should be considered as periods of increased risk for cholera outbreaks. We recommend that cholera control guidelines from local to international levels recognize drought periods as higher risk times, warranting increased vigilance to prevent and control cholera outbreaks, as well as floods. Research on how a lack of water, hygiene, and crowding may affect cholera transmission during droughts and floods is needed to better understand the drivers that are perpetuating this deadly disease.

## Supplementary Material

Supplemental Figures and Tables
