# Supplementary material for: Exploring Droughts and Floods and Their Association with Cholera Outbreaks in Sub-Saharan Africa: A Register-Based Ecological Study from 1990 to 2010
Source: Am J Trop Med Hyg. 2018 Mar 5;98(5):1269–74. doi: 10.4269/ajtmh.17-0778 (PMC5953376; doi:10.4269/ajtmh.17-0778)
Supplement: Supplementary file 1 [file tpmd170778.SD1.pdf]

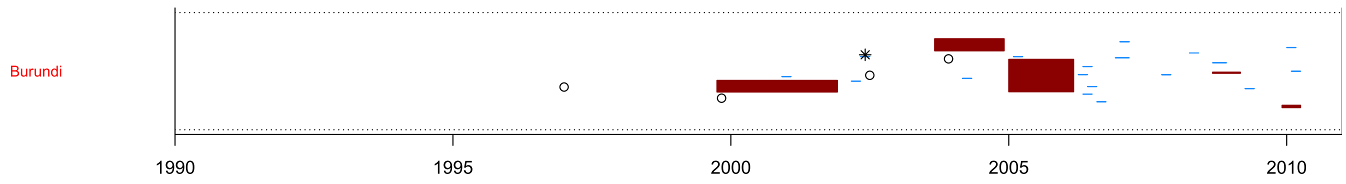

Supplemental Figure 1. Illustration of risk time attribution in Burundi.

Supplemental Table 1
Definitions

Terms	Definitions (from www.emdat.be/explanatory-notes)
EM-DAT's definition of a disaster	*"An event in which ten or more people have died, and/or hundreds or more people are affected, and/or state of emergency declared, and/or call for international assistance."*
EM-DAT's definition of a drought	"*Long lasting event; triggered by lack of precipitation. A drought is an extended period of time characterised by a deficiency in a region's water supply that is the result of constantly below average precipitation. A drought can lead to losses to agriculture, affect inland navigation and hydropower plants, and cause a lack of drinking water and famine*."
EM-DAT's definition of flood	"*Significant rise of water level in a stream, lake, reservoir or coastal region*."
EM-DAT's definition of cholera outbreak	No definition by EM-DAT
Reputable source	MSF, promedmail.org, reliefweb.org, IFRC, WHO/PAHO, UNICEF, IRIN News, and peer-reviewed journals
Definition of a cholera endemic country by Ali et al.[19] the definition origins from work by the WHO Strategic Advisory group of Experts on Vaccines and Immunization (SAGE).[20]	We considered endemicity to begin when the first outbreak began during a 5-year period where cholera outbreaks were witnessed during three or more years. Likewise, endemicity was considered to have ended 5 years after the start of the third to last cholera outbreak within a 5-year period unless the conditions for endemicity continued thereafter. If one or more cholera outbreaks were registered from 2006 to 2010 that did not meet the endemicity criteria within the dataset, a Google search was performed to see if cholera cases or outbreaks occurred after the study period (2011–2014) to determine if the outbreaks occurred during an endemic period.

WHO = World Health Organization.

Supplemental Table 2
Overview of missing day, month, and year values for the start and end dates of the events

	Number of registered events missing a start date			Number of registered events missing an end date		
	Day	Month	Year	Day	Month	Year
Drought (118)	118 (100%)	18 (15%)	0	118 (100%)	85 (72%)	0
Flood (515)	102 (20%)	0	0	110 (21%)	0	0
Cholera (276)	116 (42%)	4 (1%)	0	–	–	–

Supplemental Table 3

Equations for calculating the incidence rate of cholera during droughts and floods

	Equation	Calculation example for droughts
Incidence rate for cholera outbreaks during droughts and floods	$IR_d = \frac{\sum_{1990}^{2010} c_d}{\sum_{1990}^{2010} a_d \times T_d}$ $IR_f = \frac{\sum_{1990}^{2010} c_f}{\sum_{1990}^{2010} a_f \times T_f}$	$IR_d = \frac{35}{31.1} = 1.1$
Incidence rate for cholera outbreaks during drought/flood-free periods	$IR_n = \frac{\sum_{1990}^{2010} c_n}{\sum_{1990}^{2010} a_n \times T_n - ([a_d \times T_d] + [a_f \times T_f])}$	$IR_n = \frac{217}{861 - 31.1 - 0.9} = 0.26$
Relative incidence rate for drought and floods	$IRR_d = \frac{IR_d}{IR_n}$ $IRR_f = \frac{IR_f}{IR_n}$	$IRR_d = \frac{1.1}{0.26} = 4.3$

I_d and I_f are the incidence of cholera during a drought period and a flood period, respectively, c_d and c_f are the number of cholera outbreaks that were matched by location to a drought and a flood, respectively, a_d and a_f are the affected individuals (as recorded in EMDAT) of all droughts and floods, respectively, and T_d and T_f are the duration of the droughts and floods, respectively. I_n is the incidence of cholera during non-drought, non-flood periods, c_n is the cholera outbreaks that were not matched by time and/or location to a drought or flood, a_n is the total population of the study countries (World Bank data), and T_n is the duration of the study period. Definitions for a_d, a_f, T_d, and T_f can be found in Figure 1.

Supplemental Table 4

Test of effect measure modification by endemicity

	Cholera outbreaks that began during the disaster	Number of disasters	Risk (cholera outbreak/ disaster)	Risk time (weighted country-years)	Incidence rate (cholera outbreaks/ weighted country-years)	Cholera outbreaks beginning during drought/flood-free periods	Drought/flood-free time (weighted country-years)	Incidence rate (cholera outbreaks/ weighted country-years)	Incidence rate ratio (95% confidence interval)
Drought									
Endemic	24	45	0.53	8.9	2.7	126	237	0.5	5.1 (3.3–7.9)
Non-endemic	11	73	0.15	22.2	0.5	91	592	0.2	3.2 (1.7–6.0)
Test for different incidence rate ratios, *P* value									0.24
Flood									
Endemic	25	216	0.12	0.5	45	126	237	0.5	86 (56–131)
Non-endemic	9	299	0.03	0.3	26	91	592	0.2	168 (85–334)
Test for different incidence rate ratios, *P* value									0.10
